# Case Report: Oculomotor Palsy With Cyclic Spasms in a Patient With Charcot-Marie-Tooth Disease Type 1

**DOI:** 10.3389/fopht.2021.748589

**Published:** 2021-10-01

**Authors:** Konrad P. Weber, Christopher J. Bockisch, Klara Landau

**Affiliations:** ^1^ Department of Ophthalmology, University Hospital Zurich, Zurich, Switzerland; ^2^ Department of Neurology, University Hospital Zurich, Zurich, Switzerland; ^3^ Department of Otorhinolaryngology, University Hospital Zurich, Zurich, Switzerland

**Keywords:** oculomotor palsy, video oculography (VOG), Hess screen test, pupillometry, Charcot-Marie-Tooth disease (CMT), amblyopia, strabismus, pediatric ophthalmology

## Abstract

Oculomotor palsy with cyclic spasms is an extremely rare condition whose exact pathophysiology remains a mystery. We followed a boy from the onset of symptoms at the age of ten months until 15 years and documented the case with video oculography. In addition, he was diagnosed with hereditary motor and sensory neuropathy (Charcot-Marie-Tooth disease type 1). Although a pure coincidence cannot be ruled out, it is conceivable that the underlying demyelinating neuropathy of this patient rendered the oculomotor nerve more susceptible to damage.

## Case Report

**Figure 1:**
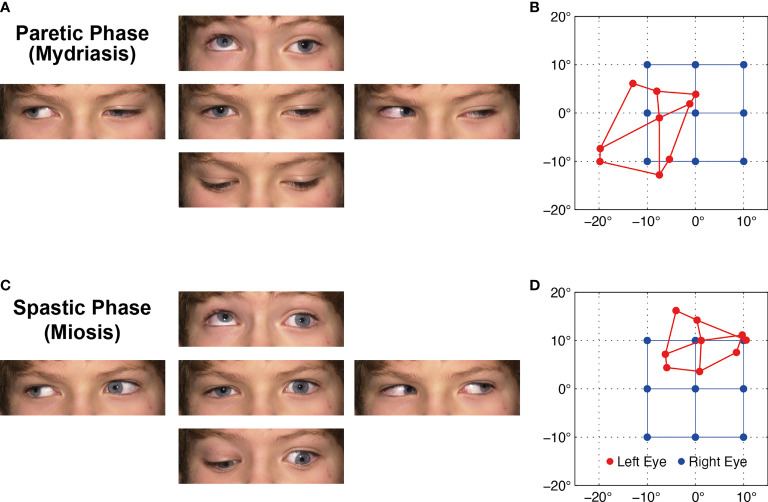
**(A)** Ocular motility during the paretic phase while the left affected eye is in mydriasis with lid ptosis. **(B)** Corresponding Hess screen test during the paretic phase with fixation of the healthy right eye (blue). The left affected eye (red) shows the typical exo- and downward deviation pattern of oculomotor nerve palsy. **(C, D)** During the spastic phase, the pupil is in miosis, the lid retracts, and the affected left eye drifts upward and inward, but the paresis persists (**Video 1**). For corresponding dynamic Hess screen recordings with binocular video oculography, see **Video 2**.

A boy developed the first signs of left oculomotor nerve paresis at the age of ten months. The paresis slowly progressed to become virtually complete by the age of four years. Five years later, the first cyclic spasms were observed: each spastic phase lasting about 20 seconds consisted of co-contraction of all affected extraocular muscles, miosis, and accommodation. The spasms were preceded by involuntary twitching of the upper lid and occurred with a periodicity of about 60 seconds (**Figures 1A, C** and **Video 1**). Follow-up at age 15 years demonstrated a very similar pattern (**Video 1**). The patient felt the lid twitches and was well aware of the cycles. A magnesium therapy trial had hardly any effect.

**Figure 2:**
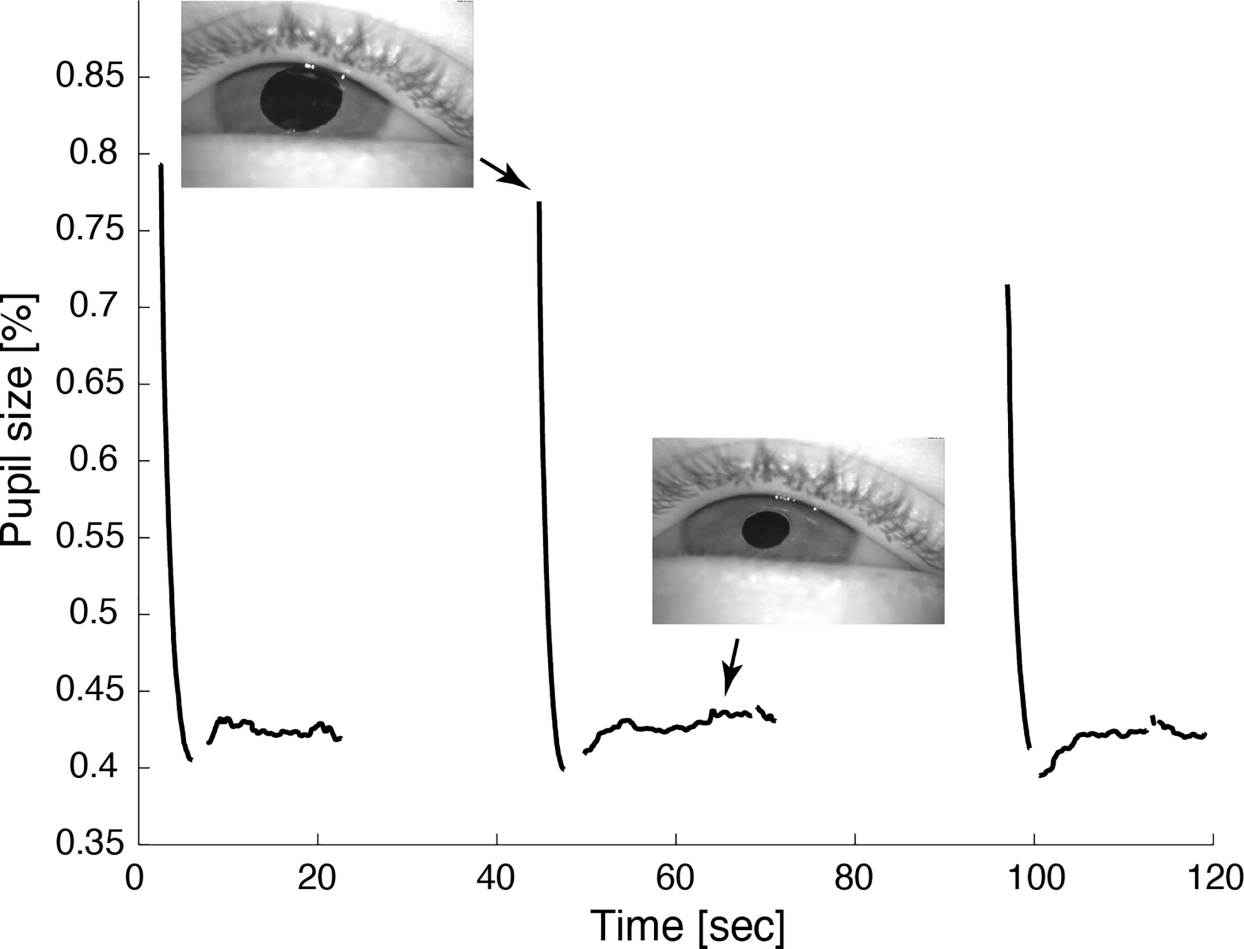
Video pupillometry demonstrates cyclic pupil contraction with a median duration of 59.8 sec. Reliable measurement of the pupil diameter was only possible while the lid was retracted during contraction of the pupil.

Hess screen testing with binocular video oculography confirmed the typical exo- and downward deviation pattern of oculomotor nerve palsy accompanied by mydriasis (**Figure 1B**). During miosis, the affected left eye drifted upward and inward, but the palsy persisted (**Figure 1D**). **Video 2** illustrates the upward and inward drift of the eye during cyclic spasms with a dynamic reconstruction of the video-oculography Hess screen recordings (1). Concurrent pupillometry timed the associated cyclic pupil contraction with a median duration of 60 sec (**Figure 2**).

The oculomotor palsy with its associated ptosis subsequently led to amblyopia with a visual acuity of 0.3-0.4 at age nine. Occlusion therapy restored the corrected visual acuity of the affected eye to 0.7 at age 15, while vision in his healthy eye remained normal at 1.25. Two consecutive cerebral MRI scans with dedicated orbits protocol and contrast enhancement, the first at age two and three quarters, the second at age nine, did not reveal any causative pathology along the left oculomotor nerve.

At age seven, the patient was also investigated for gait disturbance. Neuropediatric examination showed absent deep tendon reflexes and a reduced sense of vibration. Electroneurography demonstrated sensory and motor nerve conduction velocities of the median nerve below 38 m/sec, indicating demyelinating neuropathy. Measurements of family members revealed that his father and sister were also affected. In addition, unilateral ptosis was reported in the great-grandfather on the father’s side. However, there was no consanguinity between the parents. The patient was otherwise in good health and later attended high school. Based on these findings, the clinical diagnosis of dominant hereditary motor and sensory neuropathy (Charcot-Marie-Tooth disease type 1, CMT 1) was made. While the most frequent mutations for CMT type 1A and 1B could not be found, the diagnosis was not investigated with further genetic testing.

## Discussion

Since the first description of oculomotor palsy with cyclic spasms by Rampoldi in 1884 (2), its pathophysiology remained subject to debate. Historically, two schools of thought emerged to explain this peculiar phenomenon: the first group of authors attributed its cause to a lesion within the oculomotor nerve. In contrast, the second group suspected damage to the oculomotor nucleus in the midbrain. After careful review of 56 literature cases and examination of a case of their own, Loewenfeld and Thompson put forward a unifying theory (3): they postulated a primary lesion of the intracranial oculomotor nerve at birth or during early childhood, followed by secondary innervation changes in the oculomotor nucleus leading to cyclic spasms. Kommerell et al. were the first to provide pathophysiological evidence for this combined hypothesis (4): in one of their cases, they demonstrated chronic neurogenic changes of the levator palpebrae upon electromyography, confirmed by neurogenic muscular atrophy with signs of ongoing reinnervation upon electron microscopy. Nan et al. (5) reviewed the Chinese literature for case reports and collated 29 patients. They found a patient report with an associated Marcus Gunn phenomenon supporting the hypothesis that cyclic spasms may be due to aberrant innervation.

The underlying cause of the chronic nerve damage, however, has been poorly understood. As the symptoms usually start before the age of two (6), the syndrome has been associated with possible injury to the oculomotor nerve at birth or during infancy (3). Occasionally, intracranial lesions compressing the oculomotor nerve, for instance, a supraclinoid aneurysm, have been reported in adults (7). Miller and Lee also described two adult patients who developed oculomotor palsy with cyclic spasms after irradiation of the skull base for intracranial tumors (8). The resulting condition differs from the more common ocular motor disorder in the context of irradiation - ocular neuromyotonia.

As concurrently diagnosed in our patient, CMT is a genetically heterogeneous condition with the common feature of primary perturbation of nerve sheath myelination (9). Segmental demyelination and re-myelination of peripheral nerves lead to a characteristic reduction of nerve conduction velocity in these patients (10). Cranial nerve involvement has been reported in certain CMT subtypes. Particularly interesting in this context is a variant of CMT 1B with facial hemispasm and trigeminal neuralgia, which also have a paroxysmal pattern (11). In contrast to our patient, these symptoms appear later in life. Although we cannot rule out a pure coincidence, it is conceivable that this underlying neuropathy rendered the oculomotor nerve of our patient more susceptible to damage, leading to oculomotor palsy with cyclic spasms.

## Data Availability Statement

The raw data supporting the conclusions of this article will be made available by the authors, without undue reservation.

## Ethics Statement

Ethical review and approval was not required for the single case report in accordance with the local legislation and institutional requirements. Written informed consent was obtained from the minor’s legal guardian/next of kin, for the publication of any potentially identifiable images or data included in this article.

## Author Contributions

KW: Manuscript preparation. CB: Video oculography, figure and video preparation, manuscript revision. KL: Patient workup, manuscript revision. All authors contributed to the article and approved the submitted version.

## Conflict of Interest

The authors declare that the research was conducted in the absence of any commercial or financial relationships that could be construed as a potential conflict of interest.

## Publisher’s Note

All claims expressed in this article are solely those of the authors and do not necessarily represent those of their affiliated organizations, or those of the publisher, the editors and the reviewers. Any product that may be evaluated in this article, or claim that may be made by its manufacturer, is not guaranteed or endorsed by the publisher.
